# Bioinspired Mechanical Structures for Load Bearing and Energy Absorption: Cross-Scale Design and Fabrication Strategies

**DOI:** 10.3390/biomimetics11090672

**Published:** 2026-09-18

**Authors:** Tao Chen, Xinyu Bai, Dongdong Sun, Yi Liu, Feilong Yin, Wenda Song

**Affiliations:** 1 Electric Power Research Institute of State Grid Jilin Electric Power Co., Ltd., Changchun 130012, China; 2 School of Mechanical and Aerospace Engineering, Jilin University, Changchun 130025, China

**Keywords:** bioinspired design, cross-scale design, load-bearing structures, energy absorption, advanced manufacturing

## Abstract

Biological systems achieve efficient load bearing and energy absorption through coordinated material organization, structural geometry, and deformation mechanisms across multiple length scales. Translating these principles into engineering structures requires a framework that connects biological mechanisms with mechanical functions and manufacturable architectures. This review examines bioinspired mechanical structures from the coupled perspectives of cross-scale design and fabrication. The design discussion is organized into four routes according to their primary mechanical roles. Internal material architectures and macroscopic structural arrangements are discussed in relation to load transfer, stress redistribution, and structural stability. Multilayer configurations and lattice-filled architectures are analyzed with emphasis on progressive deformation, failure regulation, and energy absorption. The review then compares biomineralization, self-assembly, freeze casting, and additive manufacturing in terms of accessible length scales, material compatibility, geometric freedom, and structural fidelity. Particular attention is given to the interactions among material distribution, load paths, deformation modes, and manufacturing defects. Current challenges include quantitative extraction of biological design principles, cross-scale integration, discrepancies between designed and as-built structures, and limited validation under realistic loading conditions. Future research should combine data-driven inverse design, multimaterial fabrication, in situ characterization, and defect-informed modeling. This review provides a function-oriented framework for developing lightweight bioinspired structures with reliable load-bearing and energy absorption performance.

## 1. Introduction

Natural biological structures have evolved to provide efficient load bearing and energy absorption with limited material use [1,2]. These mechanical advantages do not arise solely from the intrinsic properties of their constituents. The spatial distribution of constituents, interfacial architecture, and structural geometry jointly govern load transfer, damage evolution, and energy dissipation. Shells [3], bones [4], exoskeletons [5], honeycomb structures [6], and fibrous systems [7] provide diverse biological models for reconciling low weight with strength, fracture toughness, and damage tolerance. As the mechanics of biological structures have become better understood, bioinspired design has progressed from the direct replication of external shapes and local features to the engineering translation of underlying design principles [8]. Bioinspired mechanical structures have consequently emerged as an interdisciplinary research field spanning materials science, structural mechanics, and advanced manufacturing.

Growing demands in aerospace, transportation, protective engineering, and biomedical applications have increased the need for lightweight structures that combine reliable load bearing with impact protection [9]. Here, load-bearing performance refers to the ability to sustain service loads while maintaining acceptable deformation and structural integrity. It is assessed through stiffness, strength, and resistance to instability under the relevant loading conditions [10]. Energy absorption refers to the mechanical work taken up by a structure during deformation. For impact protection, the objective is to dissipate energy through controlled deformation while limiting transmitted peak forces. Relevant indicators include absorbed energy, specific energy absorption, and peak force, supplemented by plateau stress and densification strain for cellular structures under compression [11]. Bioinspired load-bearing designs therefore emphasize efficient load transfer and structural stability, while energy-absorbing designs exploit mechanisms such as progressive collapse, interfacial sliding, and distributed damage. These functions can coexist within one architecture, although increased stiffness or strength alone does not ensure improved impact protection. Their distinct mechanical objectives provide the functional basis for organizing the design strategies reviewed here.

Both load-bearing performance and energy absorption can be regulated across different spatial scales. At the material scale, phase distribution, interfacial architecture, fiber orientation, and spatial gradients govern local load transfer and crack propagation. The hierarchical organization of dental enamel provides a representative example of how microstructural arrangement can improve strength and damage tolerance [12]. At the structural scale, cellular arrays, ribs, partitions, layered configurations, and graded geometries can redistribute loads and alter failure sequences [13,14]. Once the external geometry and boundary conditions are specified, lattice cells and internal infill topologies provide an additional means of controlling local stress, collapse behavior, and effective mechanical response [15,16]. These design domains do not represent successive levels within a single structural hierarchy. In this review, cross-scale design refers to the extension of bioinspired design from material microstructures to macroscopic structural configurations and internal infill topologies.

Translating these design principles into engineered structures requires fabrication methods that can reproduce the intended material and geometric features. Material-scale designs require precise control over constituent distribution, orientation, and interfacial bonding. Macroscopic configurations and internal infill topologies place additional demands on geometric accuracy, feature size, and defect control. Additive manufacturing enables the direct fabrication of complex internal geometries and spatially graded material distributions [17,18]. Previous research has shown that this technology is being explored for small spacecraft, load-bearing components, and broader space applications [19,20]. The return of the first metal specimen printed aboard the International Space Station for ground testing further demonstrates the progress of in-space additive manufacturing [21]. These developments allow bioinspired designs to be evaluated as manufacturable engineering structures rather than as geometric concepts alone.

Recent studies illustrate how cross-scale design and advanced fabrication can improve both load-bearing performance and energy absorption. Prihar et al. used robotic additive manufacturing to produce double-Bouligand concrete inspired by coelacanth scales. The resulting architecture increased fracture toughness and promoted a less brittle damage response [22]. Wen et al. introduced a spatial gradient of stiff and compliant phases into a double-twisted Bouligand architecture. The printed structures exhibited improved protection over a wide range of impact velocities [23]. He et al. integrated material architecture, structural topology, and additive manufacturing in aluminum-based metamaterials. Their approach combined low density with high strength, fracture resistance, and energy absorption [24]. The reported improvements arose from the combined control of material organization, structural configuration, deformation pathways, and fabrication quality.

Previous reviews have examined biological design principles for lightweighting [9], the mechanical roles of structural elements [13], and lattice architectures for impact and energy absorption [11]. These contributions establish important relationships between biological structures and mechanical performance. However, structural labels such as layered, graded, or cellular do not by themselves distinguish the roles of load transfer, damage resistance, and collapse regulation. The present review organizes studies jointly by their primary mechanical objective and the structural domain targeted by the design. This allows different biological models and geometries to be compared through their governing mechanisms, while recognizing that one feature may contribute to both load bearing and energy absorption. The analysis further examines how these mechanisms cooperate or compete across structural domains and how fabrication resolution, interfacial continuity, and manufacturing defects affect their realization. The distinctive contribution of this framework is the combined assessment of mechanical roles, cross-scale interactions, and manufacturing requirements. Reported mechanical outcomes are interpreted in relation to loading conditions, reference configurations, and fabrication constraints to clarify the basis and limitations of comparisons between studies. To support a comparison based on mechanical function, the literature for this review was identified through searches of Web of Science Core Collection and Scopus, supplemented by examination of reference lists in relevant reviews and primary studies. Searches combined “bioinspired” or “biomimetic” with “load bearing”, “energy absorption”, “toughness”, “impact resistance”, “hierarchical structures”, “graded structures”, “cellular structures”, “lattice”, and “triply periodic minimal surface (TPMS)”. Additional searches used “biomineralization”, “self-assembly”, “freeze casting”, and “additive manufacturing” to identify relevant fabrication studies. The principal publication window was 2020–2026, with particular emphasis on developments from 2023 to 2026. Earlier foundational publications were retained to establish biological mechanisms and manufacturing principles.

**Figure 1:**
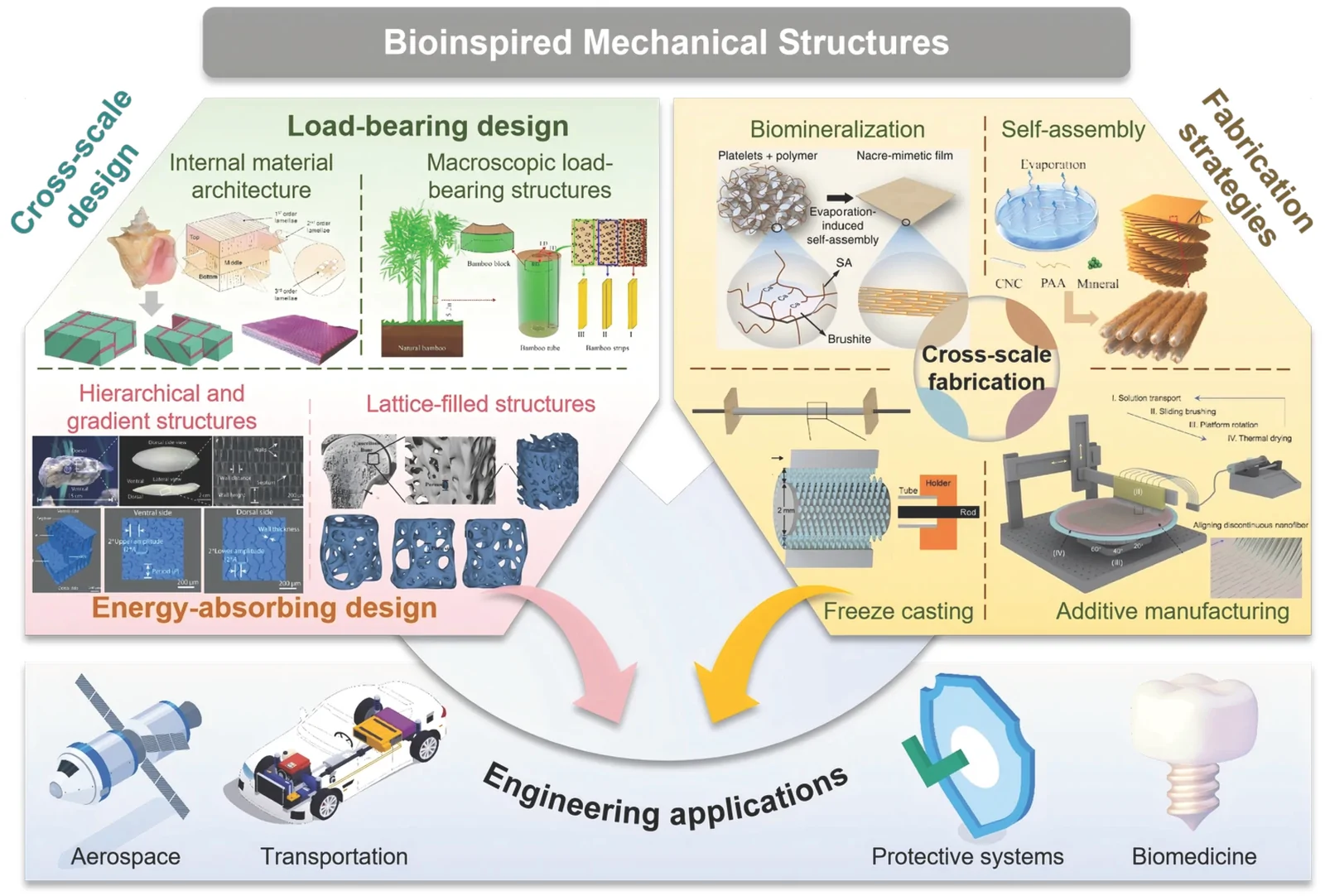
Cross-scale design and fabrication strategies for bioinspired mechanical structures for load bearing and energy absorption. Reproduced with permission from [2], copyright 2025 Elsevier.

Accordingly, this review examines cross-scale design and fabrication strategies for bioinspired mechanical structures with load bearing and energy absorption as the two functional objectives (Figure 1). Primary studies were included when they connected biological or engineered structural features with mechanical behavior, or demonstrated fabrication capabilities relevant to material organization, geometric control, and structural fidelity. Eligible evidence included experimental characterization, numerical analysis, and documented fabrication outcomes. Duplicate records were removed, followed by title and abstract screening and full-text assessment. Selection prioritized mechanistic relevance, sufficient structural and methodological information, and representation across the design and fabrication routes. Studies devoted solely to actuation, sensing, optical response, or surface functionality were excluded from the comparative synthesis unless they directly informed the mechanical functions or fabrication requirements considered here. Reviews, books, standards, and agency reports provided supporting context. The review draws on approximately 110 reference sources in total. The discussion of load-bearing design examines how constituent distribution and interfacial architecture govern load transfer and damage resistance at the material scale. It also considers how macroscopic structural configurations influence load paths, stress redistribution, and structural stability. The discussion of energy absorption addresses progressive failure in layered, compartmentalized, and graded structures. It further discusses how lattice cells and internal infill topologies control collapse modes, plateau response, densification, and energy absorption. Fabrication strategies based on biomineralization, self-assembly, freeze casting, and additive manufacturing are then reviewed. Finally, this review discusses cross-scale coupling, trade-offs between load-bearing performance and energy absorption, and remaining challenges in quantitative design, fabrication accuracy, and engineering validation. This review connects biological principles with engineering design and fabrication to guide the development of lightweight structures with reliable load-bearing performance and controlled energy absorption.

## 2. Cross-Scale Design of Bioinspired Mechanical Structures for Load Bearing and Energy Absorption

Biological load-bearing and protective systems often integrate several structural features. These include heterogeneous phases, interfaces, cellular arrays, layered compartments, gradients, and porous networks. A classification based only on morphology would therefore place many biological models and engineered designs in more than one category. This section instead organizes bioinspired mechanical structures according to their primary mechanical function and the structural domain targeted by the design. Material microstructural organization and macroscopic structural configurations are examined as two routes to effective load transfer and stable load bearing. Layered and graded configurations, together with internal infill topologies, are discussed in relation to progressive failure and energy absorption. Cross-scale design in this review does not imply that all structures follow a common hierarchical sequence. It describes the use of design variables across material microstructures, component-scale configurations, and internal infill topologies. The final subsection examines interactions among these design domains and the trade-offs between load-bearing performance and energy absorption.

### 2.1. Load Transfer and Damage Resistance Through Material Microstructural Organization

At the material scale, load-bearing performance depends on how stress is transmitted among constituents and across their interfaces. Bioinspired material design therefore focuses on the spatial organization of phases, interfaces, and reinforcing elements rather than on reinforcement content alone. Recent research has identified constituent arrangement, interfacial architecture, and multiscale connectivity as major sources of mechanical performance in biological and bioinspired materials [25,26,27]. Nacre, bone, spider silk, wood, and tendon provide representative biological models [28]. Their internal structures combine stiff and compliant phases with oriented fibers, confined interfaces, and gradual changes in composition or stiffness. These features redistribute local stress, extend crack paths, and delay the transition from local damage to catastrophic failure. Three recurring strategies are especially relevant to load bearing. They are continuous load-bearing networks, interfacial interlocking, and heterogeneous phase organization.

**Figure 2:**
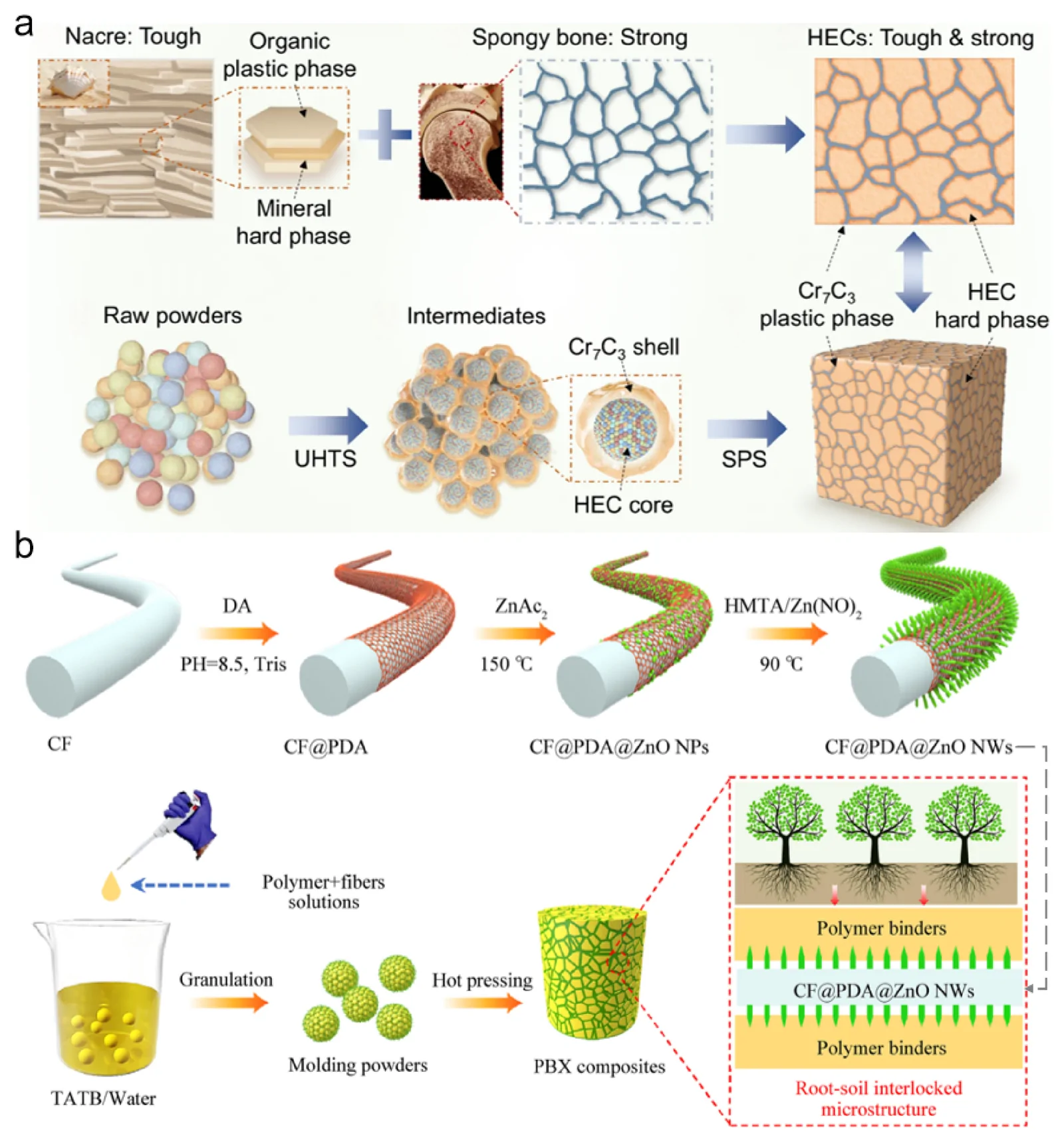
Representative bioinspired material microstructures for improving load transfer and damage resistance. (**a**) Design and fabrication of HEC/Cr_7_C_3_ all-ceramics inspired by nacre and spongy bone. A contiguous Cr_7_C_3_ plastic-phase network is formed within the predominant HEC hard phase through ultrafast high-temperature synthesis and spark plasma sintering. Reproduced with permission from [29], copyright 2025 Springer Nature. (**b**) Construction of a root–soil interlocked interface in a polymer-bonded explosive. ZnO nanowires grown in situ on polydopamine-modified carbon fibers act as root-like reinforcements embedded within the polymer binder network. Reproduced with permission from [30], copyright 2026 Elsevier.

Continuous networks create interconnected paths for stress transmission through the bulk material. Zhu et al. combined the brick-and-mortar architecture of nacre with the contiguous trabecular network of spongy bone to develop high-entropy carbide (HEC)/Cr_7_C_3_ all-ceramics [29]. As shown in Figure 2a, the plastically deformable Cr_7_C_3_ phase formed a contiguous network within the predominant HEC hard phase. Mixed raw powders were first converted into HEC/Cr_7_C_3_ core–shell intermediates by ultrafast high-temperature synthesis. The intermediates were subsequently densified by spark plasma sintering. This process produced a continuous Cr_7_C_3_ network throughout the ceramic matrix. Unlike isolated toughening particles, the connected Cr_7_C_3_ phase redistributed stress among neighboring regions and interrupted direct crack propagation. The resulting ceramic exhibited a fracture initiation toughness of 12.5 ± 1.5 MPa·m^0.5^ and a flexural strength of 613 ± 52 MPa at room temperature. It retained approximately 97% of its flexural strength at 1300 °C. The Cr_7_C_3_ phase accommodated plastic deformation through nanoscale shear bands. Unfractured Cr_7_C_3_ ligaments bridged crack faces, while the heterogeneous HEC/Cr_7_C_3_ interfaces deflected propagating cracks. The continuous network therefore supported bulk load transfer and provided distributed resistance to crack propagation.

Interfacial organization becomes critical when loads are transferred between constituents with different stiffnesses. Weak interfacial bonding produces stress concentrations and promotes premature debonding. Liu et al. introduced a root–soil interlocked microstructure into polymer-bonded explosives [30]. Figure 2b summarizes the surface modification and composite fabrication processes. A polydopamine coating was first formed on the carbon fibers. ZnO nanoparticles were then deposited as nucleation sites for the in situ growth of ZnO nanowires. The modified fibers were incorporated into a 1,3,5-triamino-2,4,6-trinitrobenzene-based formulation. Granulation produced molding powders, which were subsequently consolidated by hot pressing. The nanowire-covered fibers acted as roots embedded within the polymer binder network representing the soil. The nanowires increased the interfacial contact area and strengthened mechanical anchoring. The polydopamine coating further improved interfacial bonding. The carbon-fiber-free polymer-bonded explosive composites control exhibited a Brazilian tensile strength of 5.42 MPa and a fracture strain of 0.1% at 25 °C. Incorporating 0.4 wt% of the modified carbon fibers increased these values by 41% and 110%, respectively. Under a constant stress of 4 MPa at 30 °C, the reinforced composite also showed a 51% reduction in creep strain relative to the same carbon-fiber-free control. This example demonstrates that interface geometry and interfacial chemistry can operate together to improve load transfer and suppress interfacial failure.

Spatial heterogeneity provides another route for controlling local stress and deformation. Xie et al. developed a minimal tropoelastin-inspired model that used controlled liquid–liquid phase separation and subsequent covalent bonding to form a heterogeneous hydrogel [31]. The phase-separated structure was stabilized during the coacervate-to-hydrogel transition and reproduced important features of elastin fibrillation. The resulting matrix promoted mechanosensing in adherent stem cells. This research focused on cellular regulation rather than structural load bearing. Nevertheless, it demonstrates how molecular interactions can control the spatial distribution and connectivity of mechanically distinct regions. Similar control could reduce abrupt stiffness changes and redistribute local strain in load-bearing materials.

Continuous networks, interlocked interfaces, and heterogeneous phase distributions act at different positions along the load path. Networks redistribute stress through the bulk material. Interlocked interfaces transfer stress across constituent boundaries. Heterogeneous regions moderate local deformation and alter damage trajectories. These mechanisms can coexist within the same material. Their effectiveness depends on network continuity, phase fraction, interface strength, and defect population. An excessive compliant-phase fraction can create preferential crack paths, whereas insufficient connectivity limits stress redistribution [26,29]. Most current research optimizes one mechanism within a specific material system. Quantitative relationships between microstructural parameters and load-bearing performance remain limited. Greater attention is also needed to manufacturing defects and the stability of internal organization at larger scales. These material-scale strategies establish the local basis for efficient load transfer and damage resistance. At the component scale, their mechanical benefits also depend on how the constituent units are arranged to form continuous load paths and maintain structural stability under complex loading conditions.

### 2.2. Load-Path Regulation and Stability Through Macroscopic Array Architectures

Material-scale organization determines how loads are transferred among individual constituents. At the component scale, overall load-bearing performance is further governed by the arrangement, connection, and constraint of repeated structural units. Biological stems, cellular tissues, honeycombs, and exoskeletal plates use ordered arrays to distribute loads over large regions with limited material. These architectures provide alternative load paths and reduce the dependence of the structure on a single supporting element. They also constrain local deformation and delay the development of global instability. Cellular architectures, functional gradients, shell–core combinations, and robust geometries have therefore been identified as important biological strategies for efficient load bearing [32]. For bioinspired array architectures, the mechanical response is controlled not only by the stiffness of each unit but also by unit geometry, inter-unit connections, and spatial distribution.

Bamboo provides a representative model of a naturally distributed load-bearing array. Its vascular bundles contain stiff fiber sheaths embedded within a more compliant parenchymatous matrix. The fiber sheaths carry most of the axial load, while the surrounding parenchyma transfers stress and restrains transverse deformation. Zhang et al. reported that the distribution density of vascular bundles in *Bambusa arundinaceae* ranged from 1.98 to 4.34 mm^−2^, while the volume fraction of fiber sheaths ranged from 35.82% to 42.58% [33]. The average compressive strength, flexural strength, and flexural modulus reached 113.99 MPa, 239.07 MPa, and 17.39 GPa, respectively. These properties were positively correlated with vascular bundle density and fiber sheath volume fraction. This relationship indicates that the load-bearing capacity of bamboo does not depend only on the properties of an individual vascular bundle. It also depends on how these bundles are distributed throughout the culm wall.

**Figure 3:**
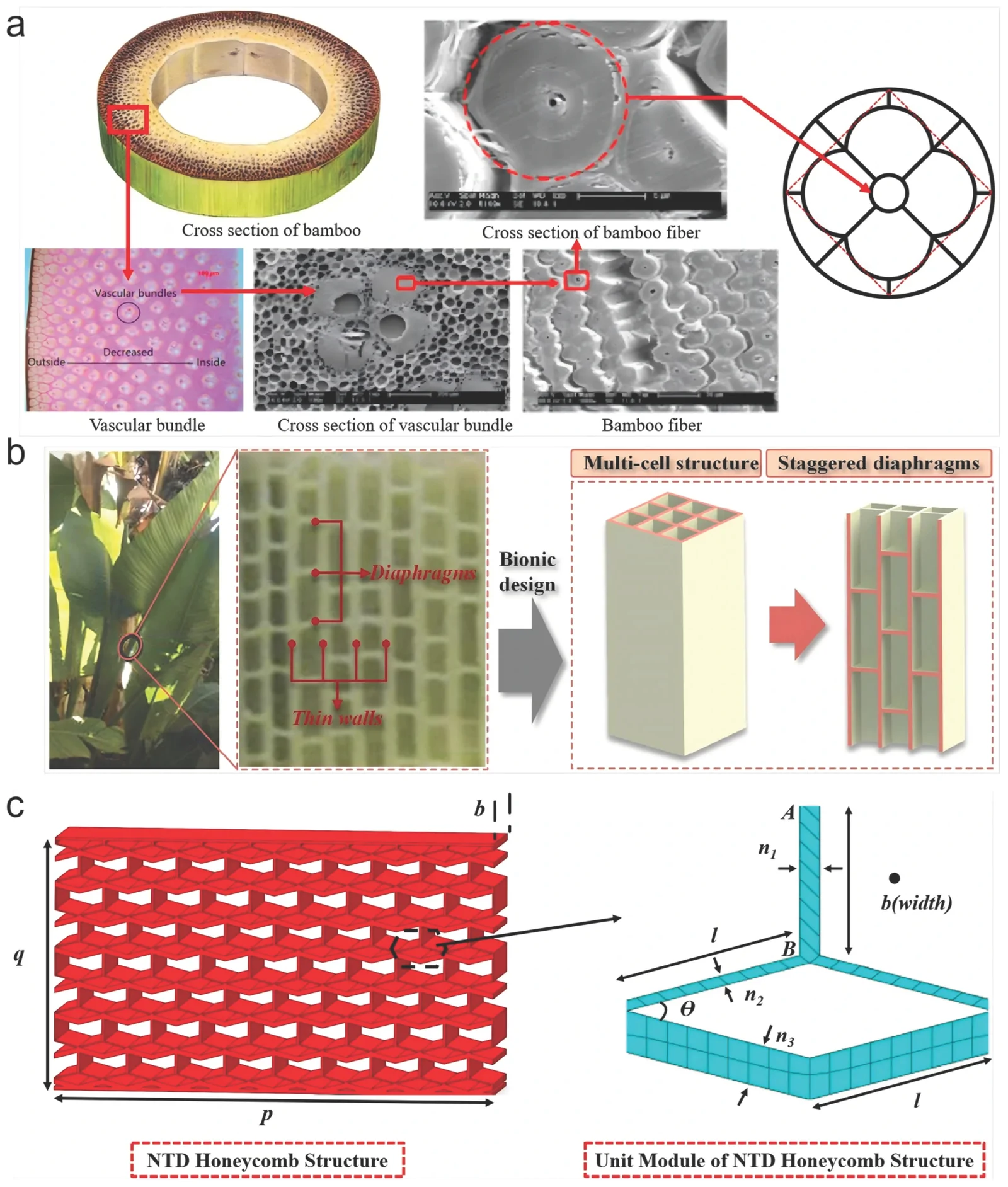
Representative macroscopic array architectures for regulating load paths and structural stability. (**a**) Structural characteristics of bamboo vascular bundles and their translation into a thin-walled tube containing a central core and radial supporting panels. Reproduced with permission from [34], copyright 2023 Elsevier. (**b**) Staggered diaphragms in the stem of the bird-of-paradise plant and the corresponding multi-cell thin-walled structure. The longitudinal walls provide the main axial load paths, while the staggered transverse diaphragms improve local constraint. Reproduced with permission from [35], copyright 2025 Elsevier. (**c**) Non-uniform wall-thickness rhombic honeycomb and its geometric unit. Members with different thicknesses provide deformable and supporting regions within the repeated array. Reproduced with permission from [36], copyright 2025 Springer Nature.

The geometric characteristics of individual biological units can be translated into engineered cross-sections. Liu et al. developed a thin-walled tube inspired by the vascular fiber sheath flaps and central through holes of bamboo vascular bundles [34]. As shown in Figure 3a, the biological features were simplified into a central circular core connected to the outer tube through radial panels. The outer tube provided global confinement, while the internal core and panels created multiple parallel load paths. This configuration distributed the axial load among several supporting regions and reduced the unsupported span of the outer wall. Parametric analysis showed that a semicircular vascular fiber sheath unit provided the most favorable response. The optimized ratio between the circular-core radius and the straight-panel length was 3:7. The connection between the internal unit and the outer wall also affected the deformation mode. Further replication of the optimized unit demonstrated that the parallel fractal arrangement provided better crashworthiness than the rotational arrangement. These results show that biological unit geometry must be considered together with its connection to the surrounding structure.

The stability of an array architecture also depends on the constraints between adjacent load-bearing walls. Tan et al. extracted the staggered diaphragm arrangement from the stem of the bird-of-paradise plant [35]. The natural stem contains longitudinal thin walls connected by transverse diaphragms at different heights. This feature was translated into a multi-cell thin-walled structure, as illustrated in Figure 3b. The longitudinal walls formed the primary axial load paths. The transverse diaphragms reduced their unsupported length and restricted lateral displacement. Staggering the diaphragms also separated the force peaks generated by different wall segments. The structures were fabricated from chopped carbon-fiber-reinforced polyamide by fused deposition modeling (FDM). Quasi-static axial compression tests were conducted at a crosshead speed of 10 mm/min to a crushing displacement of 60 mm, corresponding to two-thirds of the initial specimen height. Compared with the non-staggered diaphragm arrangement, the experimental results for the staggered-diaphragm configuration (SDA3) showed a 30.085% increase in specific energy absorption and a 45.674% reduction in the undulation of load-carrying capacity. The reduction in load fluctuation is particularly relevant to structural stability. It indicates that staggered constraints can prevent simultaneous buckling of adjacent walls and maintain a more uniform compressive response.

Spatial stiffness allocation provides another approach to controlling load transmission across an array. Du et al. developed a rhombic honeycomb with nonuniform wall thickness, as shown in Figure 3c [36]. The unit cell contained inclined members with different thicknesses and a vertical supporting member. The thin and thick inclined members had thicknesses of 0.95 and 3.78 mm, respectively, while the vertical member had a thickness of 1 mm. The angle between the inclined members was 29°. This heterogeneous configuration divided the unit cell into deformable and supporting regions. Under relatively low loads, the thinner inclined members accommodated deformation, while the thicker and vertical members preserved the main supporting path. Additional members became involved as the applied load increased. This sequential response reduced the concentration of deformation within a single region. In the proposed engineering device, four parallel units were designed to sustain an initial support load of 4333 kN. The design illustrates how wall-thickness allocation can be used to balance local compliance with global load-bearing stability.

These examples demonstrate that macroscopic array design operates through several complementary mechanisms. Cross-sectional geometry divides the applied load among parallel paths. Diaphragms and connecting walls maintain local stability and coordinate the deformation of neighboring units. Spatial variation in wall thickness directs loads toward stronger regions while retaining controlled compliance in less critical regions. Their combined effectiveness depends on unit dimensions, connection positions, relative stiffness, and array orientation. Manufacturing defects at repeated joints can also accumulate across the structure and alter the intended load path. Most existing research remains focused on axial compression or a single impact direction. The stability of bioinspired arrays under bending, torsion, combined loading, and cyclic loading has received less attention. The effects of unit-size variation and local defects are also not fully understood. Designs optimized for one loading direction may lose their advantage when the load direction changes. Future array architectures should therefore incorporate directional reinforcement, structural redundancy, and heterogeneous unit distributions. These features are essential for maintaining continuous load paths under complex loading conditions.

Macroscopic arrays are effective when their internal load paths remain stable. Under severe impact, however, local buckling and structural failure may become unavoidable. Mechanical performance then depends on where deformation initiates and how damage propagates through the structure. Layered and graded architectures provide a means of distributing this damage across successive regions and maintaining a controlled response during progressive collapse.

### 2.3. Progressive Energy Absorption Through Layered and Graded Architectures

The array architectures discussed above use cooperative load paths to resist instability under service loads. When a structure is exposed to crushing or impact, however, protection requires a different mechanical response. The structure must accommodate large deformation without transmitting an excessive peak force or losing its resistance abruptly. Biological systems often achieve this response through stacked layers, partitioned chambers, spatial gradients, and regions with different stiffness. Their engineering analogues aim to delay deformation localization, extend the crushing plateau, and increase energy absorption at low mass. The key design variable is therefore not the number of layers alone. It is the order in which individual layers activate, interact, and fail [37,38].

**Figure 4:**
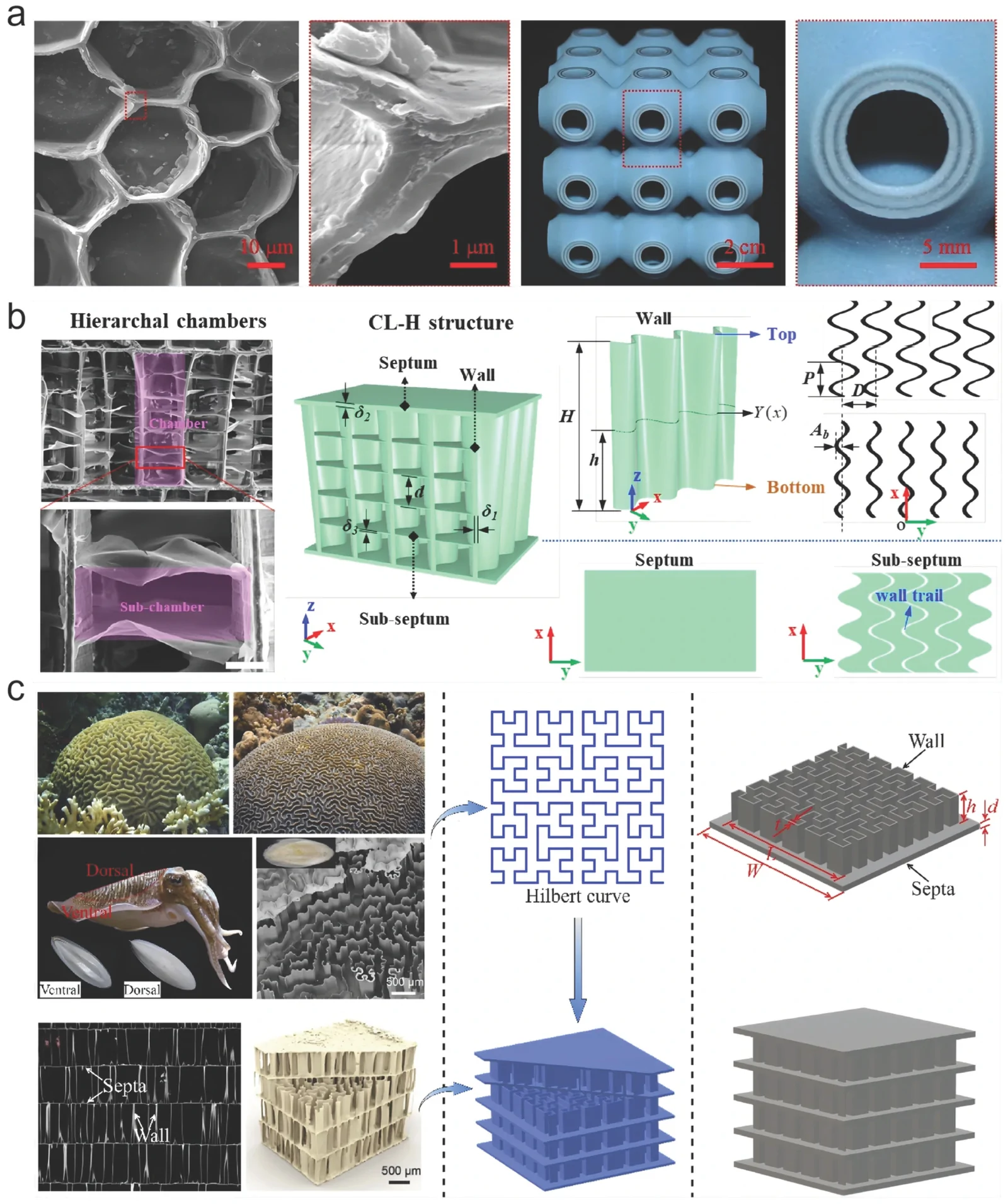
Representative layered and graded bioinspired architectures for progressive energy absorption. (**a**) Cork microstructure and a five-layer cellular composite inspired by its multilayered cell walls, with alternating hard and soft phases. Reproduced with permission from [39], copyright 2020 Elsevier. (**b**) Natural cuttlebone chambers and the corresponding cuttlebone-like hierarchical cellular structure. Walls, septa, and sub-septa divide the structure into coupled collapse units. Reproduced with permission from [40], copyright 2023 Elsevier. (**c**) Biological prototypes and the design route for the brain-coral-inspired and cuttlebone-inspired multilayer sandwich structure. The Hilbert space-filling curve defines the cellular core, while the stacked layers regulate axial collapse. Reproduced with permission from [42], copyright 2024 SAGE Publications.

Material contrast can establish the first level of sequential response. Inspired by the multiscale cellular organization of cork, Jiang et al. designed a multilayer cellular composite composed of alternating hard, brittle phases and soft, flexible phases (Figure 4a). The hard phase supported the applied load, while the soft phase redistributed strain and stored recoverable elastic energy. Under quasi-static uniaxial compression at a nominal strain rate of 5.5 × 10^−4^ s^−1^ (2.5 mm/min), the composite exhibited approximately four times the volumetric energy absorption of a single-material VeroWhitePlus reference at 70% strain. Both structures had the same Schwarz P geometry and approximately equal mass; energy absorption was calculated from the stress–strain integral without mass normalization. The multilayer architecture also promoted a more uniform strain distribution and progressive failure. Cyclic tests demonstrated shape recovery after compression to 40% strain, with heating used to accelerate recovery between cycles [39]. These findings illustrate how constituent contrast and phase fraction govern the combination of energy absorption and recoverability.

Hierarchical chambering provides a distinct geometric route. Cui et al. reproduced the walls, septa, and sub-septa of cuttlebone in hierarchical cellular structures fabricated by digital light processing (DLP) (Figure 4b). Under quasi-static axial compression at 1 mm/min, the hierarchical configuration CL-H4 achieved a specific energy absorption of 2.941 J/g, compared with 0.424 J/g for the non-hierarchical CL reference, both at a designed relative density of 15%. Absorbed energy was evaluated up to densification onset for CL-H4 and complete loss of load-bearing capacity for CL [40]. The sub-septa restricted buckling to individual sub-chambers and promoted progressive collapse. Chen et al. further developed a cuttlebone-inspired square honeycomb (S-CIH) with sinusoidal walls and an amplitude gradient. Under quasi-static out-of-plane compression at 3 mm/min, the DLP-printed G2T3B1 configuration achieved an experimental specific energy absorption of 8.859 J/g, evaluated up to densification onset. This exceeded the regular square honeycomb (S-RH, G0T0B0) and the best-performing uniform-amplitude sinusoidal square honeycomb (S-SH, G0T1B1) references by 41.4% and 74.4%, respectively. The comparison used a designed relative density of 19.5% and specimen masses of 5.8–5.9 g [41]. These studies demonstrate how chamber partitions and wall-amplitude gradients regulate buckling initiation and failure propagation during crushing.

Gradients can further program the collapse sequence at the component scale. Zhang et al. combined the surface pattern of brain coral with the layered chambers of cuttlebone to develop a multilayer sandwich structure with a Hilbert core, as shown in Figure 4c. The four-layer configuration was selected for gradient optimization. The finite element model was validated against quasi-static axial compression experiments at 5 mm/min. Numerical analyses used an accelerated platen speed of 1 m/s, with kinetic energy below 0.1% of internal energy supporting the quasi-static approximation. Nominal strain rates were not reported. Compared with the four-layer baseline with uniform 10 mm layer heights and 0.5 mm wall thickness, the optimized knee-point design increased specific energy absorption from 4.45 to 5.42 J/g (21.8%) and reduced the initial peak force from 18.1 to 4.96 kN (72.6%) [42]. The optimized design retained uniform layer heights while introducing a wall-thickness gradient, promoting progressive buckling from thinner to thicker regions within each layer.

Hybrid cores offer another route to distribute deformation. Ghanbari and Panirani combined a beetle-forewing-inspired triangular grid with bamboo-inspired bi-tubular reinforcements at grid junctions. In finite element simulations of dynamic axial crushing at 100 m/s (estimated nominal strain rate of 1.0 × 10^3^ s^−1^), the optimized hybrid achieved a specific energy absorption of 188.13 kJ/kg at 75% compression, compared with 142.47 kJ/kg for the conventional triangular grid without tubular reinforcements. Both designs had a mass of 0.6 kg, and specific energy absorption was calculated by dividing the force-displacement integral by structural mass, giving an increase of approximately 32%. The hybrid core was subsequently evaluated in sandwich-panel simulations under blast loading [43]. The grid walls and tubular reinforcements provide complementary sites for plastic deformation during dynamic crushing.

Hierarchical multi-cell tubes apply a similar principle to thin-walled energy absorbers. Bioinspired bi-tubular and branched multi-cell configurations place smaller cells within selected regions of a larger cross-section. These cells support neighboring walls, promote distributed folding, and regulate the mean crushing force. Recent studies have shown that local subdivision can improve the specific energy absorption while retaining a controllable crushing response [44,45]. The benefit is not monotonic. Excessive subdivision can increase structural mass, raise the initial peak force, or cause early interaction between adjacent folds. The hierarchy level, branch position, and wall thickness must therefore be evaluated together under axial, oblique, and dynamic loading.

These studies reveal three primary mechanisms for macroscopic energy absorption. Mechanical contrast redistributes strain between adjacent layers. Hierarchical chambers determine where collapse begins and how it spreads. Gradients and hybrid cores tune the timing and magnitude of successive force peaks. Their effectiveness depends on both geometry and constituent behavior. Most reported designs are still evaluated under quasi-static axial compression or a single impact condition. Oblique impact, repeated loading, and rate-dependent material behavior can change the intended collapse sequence. A further concern is the frequent use of ductile or elastomeric polymers to reproduce mineralized biological structures. Cuttlebone dissipates energy partly through brittle damage, whereas many engineering analogues rely on elastic buckling or plastic folding. Direct comparisons can therefore obscure the contribution of geometry. Future studies should separate structural and constitutive effects. The initial peak force, mean crushing force, specific energy absorption, and crush force efficiency should also be reported under consistent mass and boundary conditions. Layering and grading program the order of collapse across a component. The response of each layer still depends on the architecture placed inside its cavities. This shifts the design focus toward the topology, relative density, and spatial distribution of the internal cellular network.

### 2.4. Energy Absorption Through Internal Lattice and TPMS Architectures

Layered and graded architectures regulate collapse across the thickness or length of a component. The deformation inside each layer is governed by the geometry of its porous core. Internal lattices convert a solid volume into a network of struts, plates, or continuous surfaces. Under compression, these elements deform through bending, stretching, buckling, contact, and fracture. Their deformation sequence governs the initial peak stress, plateau stress, densification strain, and absorbed energy. Biological porous skeletons provide useful models because they combine low density with distributed load transfer and controlled instability. These principles have driven bioinspired lattice design toward hierarchical, auxetic, hybrid, and spatially heterogeneous architectures [46,47].

**Figure 5:**
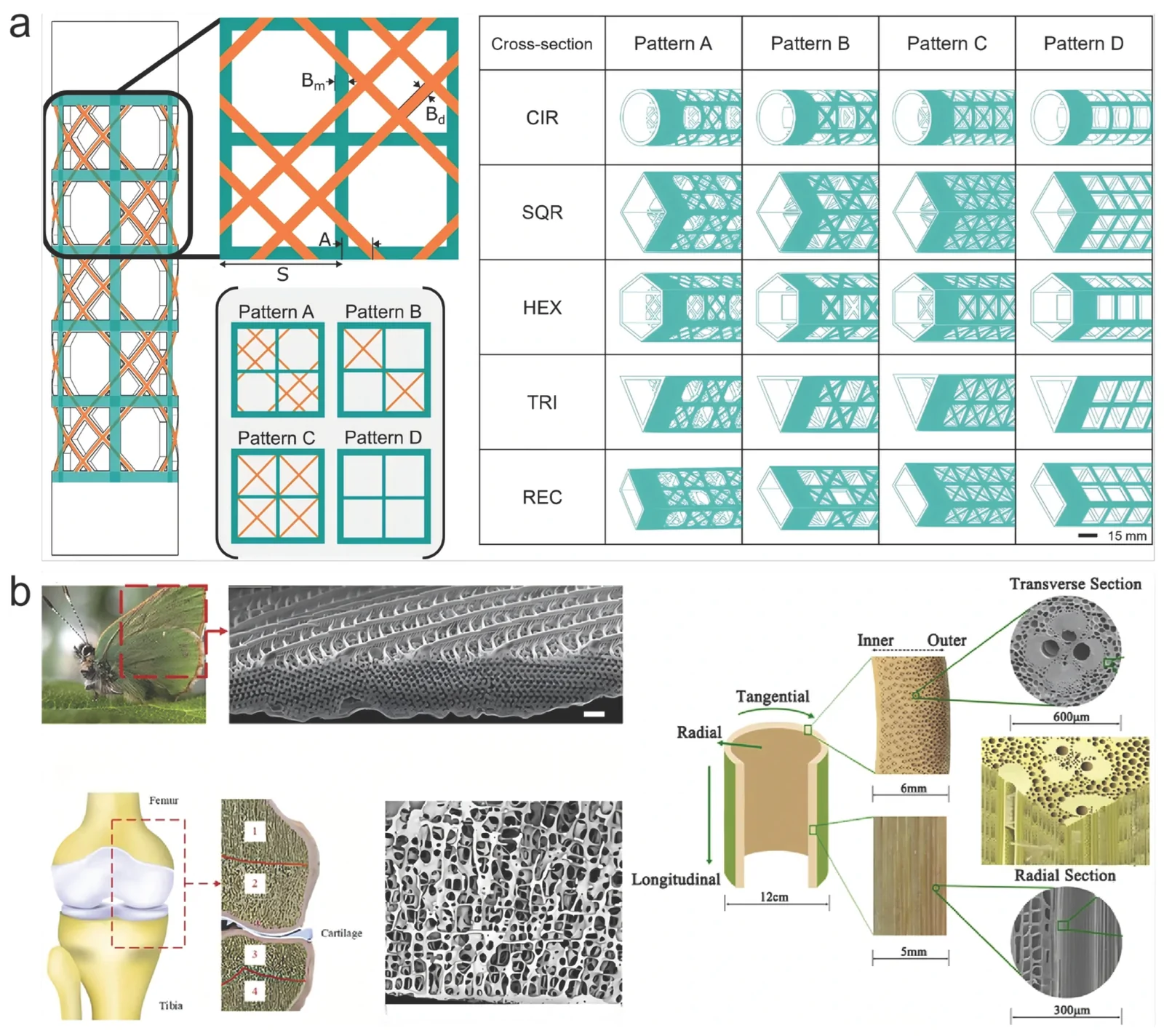
Biological inspiration and internal lattice design for energy absorption. (**a**) Design variables for sea glass sponge-inspired tubular lattices. The reinforced unit-cell patterns are combined with circular, square, hexagonal, triangular, and rectangular cross-sections. Reproduced with permission from [48], copyright 2025 Elsevier. (**b**) Naturally occurring hybrid cellular architectures in a butterfly wing, bone tissues, and bamboo. These biological systems combine pores, ribs, struts, plates, and spatially heterogeneous regions within a single architecture. Reproduced with permission from [50], copyright 2025 John Wiley and Sons.

Sea glass sponges possess tubular skeletons reinforced by orthogonal grids and diagonal braces. Chen et al. transferred this organization into three-dimensional tubular lattices. Five cross-sectional shapes and four reinforcement patterns were investigated, as shown in Figure 5a. The sponge-inspired design with a hexagonal cross-section increased the buckling strength by 74.9% relative to the corresponding non-reinforced design. The circular configuration increased elastic energy absorption by 90.8%. The diagonal members divided the primary cells into smaller triangular regions. They also shortened the unsupported length between adjacent nodes. These features improved stress distribution and delayed localized buckling [48]. The crossed skeletal network of deep-sea hexactinellid sponges has also inspired auxetic lattice design. Ma et al. developed a bioinspired lattice that exhibited a negative Poisson’s ratio under compression. The design outperformed the conventional re-entrant hexagonal honeycomb in stiffness and energy absorption. The coupled rotation and deformation of its crossed members also produced a broader auxetic strain range. Member thickness and spacing provided additional control over the compressive response [49]. This work shows that auxeticity and energy absorption can be combined without relying on conventional re-entrant cells.

Natural porous tissues rarely consist of one cell type repeated throughout the entire volume. Khan et al. identified several naturally occurring hybrid architectures, as shown in Figure 5b. The butterfly wing combines porous gyroid-like regions with surface ribs. Bone contains interconnected struts and plates with local variations in density and orientation. Bamboo integrates vessels, fibers, and matrix regions across its radial and tangential directions. These biological systems assign different mechanical roles to distinct structural elements. They therefore provide a basis for combining several lattice topologies within the same engineering structure [50].

Engineering hybridization can combine complementary deformation mechanisms. Naji et al. integrated simple cubic or face-centered cubic plate lattices with Schwarz Primitive, Gyroid, and Diamond TPMS lattices. Six hybrid configurations were fabricated and tested. The simple cubic and TPMS combinations showed the largest improvements. Their stiffness, strength, and energy absorption increased by up to 171%, 125%, and 117%, respectively, compared with the corresponding TPMS lattices. The Diamond and simple cubic hybrid also maintained a high plateau stress after yielding [51]. These results show that hybrid performance is not a simple average of the parent lattices. The spatial compatibility between different topologies determines whether their deformation mechanisms act cooperatively.

Network entanglement provides an additional mechanism for coupling load transfer and energy dissipation. Inspired by double-network hydrogels, Surjadi et al. combined stiff monolithic trusses with compliant woven networks, demonstrating complementary stiffness and stretchability under uniaxial tension [52]. Friction between entangled networks contributed to energy dissipation, while deliberately introduced internal defects helped distribute failure. Carton et al. developed a graph-based framework for three-dimensional woven metamaterials, using topology and fiber geometry to tune large deformation and failure patterns [53]. Tensile experiments and modeling highlighted the contributions of fiber entanglement and frictional contact to energy dissipation. These studies broaden the available design mechanisms for internal lattices, although their performance under compressive crushing and impact requires separate validation.

TPMS lattices are especially suitable for internal energy-absorbing cores. They are defined by implicit mathematical functions and contain smooth, continuous surfaces. Their geometry reduces sharp nodal transitions and promotes stress redistribution. The mechanical response can be adjusted through the level-set parameter, wall thickness, cell size, orientation, and relative density. Gradient and heterogeneous TPMS designs provide further control over the location and sequence of collapse. However, current metallic TPMS studies still focus mainly on uniform porosity and single topologies. The effects of heterogeneous topology, complex external geometry, and spatially varying parameters remain less established [54].

Directional organization provides another control variable. Boda et al. developed nested lattices inspired by cortical bone osteons, golden spirals, and fractal patterns. Repeated X-shaped members were arranged with different nesting orders and orientations. These variables enabled a continuous transition from near-isotropic behavior to controlled anisotropy [55]. Energy absorption was not the primary target of that study. However, directional stiffness affects the onset and propagation of collapse under oblique or multiaxial loading. Nested organization may therefore provide a useful basis for designing absorbers with direction-dependent responses.

Local heterogeneity can also be introduced without changing the global lattice family. Zhao et al. shifted the central nodes of body-centered cubic lattices to imitate point defects in crystalline materials. Depending on the node position, the elastic modulus increased by 41.5–154.1%, the yield strength increased by 40.2–110.8%, and energy absorption increased by 96.2–245.2%. Several configurations also exhibited a negative Poisson’s ratio [56]. The node offset changed the orientation of the surrounding struts and redistributed stress within the unit cell. This finding shows that a controlled geometric defect can serve as a design variable rather than an unavoidable structural weakness.

Internal lattice architectures improve energy absorption through three closely related mechanisms. Topological reinforcement delays premature buckling. Hybridization combines distinct load-transfer and collapse modes. Spatial heterogeneity controls where deformation begins and how it propagates. Their relative contributions cannot be evaluated from geometry alone. The base material, relative density, specimen size, loading rate, and densification criterion also affect the measured response.

Direct comparison among existing studies remains difficult because these parameters are rarely consistent. Performance improvements calculated against different reference lattices should not be treated as equivalent. Thin struts and curved surfaces are also sensitive to dimensional deviations, surface roughness, trapped powder, and partially fused material. These defects can change the intended failure mode and reduce the usable deformation range. Future studies should compare lattice designs at equal mass and relative density. The loading history and densification criterion should also be reported clearly. Dynamic impact, oblique loading, repeated compression, and multiaxial loading require further experimental validation.

Layered and graded architectures determine when different regions of a component collapse. Internal lattices determine how each region deforms and dissipates energy. Their integration offers a route toward lightweight absorbers with a low initial peak force, a stable crushing plateau, and high specific energy absorption. Realizing this potential requires the biological model, structural hierarchy, internal topology, material behavior, and fabrication process to be considered within a unified cross-scale design framework.

### 2.5. Cross-Scale Coupling of Load-Bearing and Energy-Absorbing Mechanisms

Cross-scale coupling depends on whether mechanisms in different structural regions remain compatible as loading increases. A structure designed for both load support and overload absorption must retain sufficient stiffness during service while allowing stable local deformation before global failure. Table 1 summarizes representative mechanisms, mechanical outcomes, and comparison limitations that inform this assessment.

The principal trade-offs concern stiffness contrast and material allocation. Compliant phases can redistribute strain and improve recoverability while reducing load-bearing stiffness [39]. At fixed material volume, additional chamber partitions require material redistribution and can reduce the thickness of primary load-bearing walls [40]. Hierarchy should therefore be selected together with local deformation thresholds and wall dimensions. The resulting deformation sequence should preserve load transfer through regions that have not yet failed. Comparisons with parent and single-feature structures under matched mass and loading conditions can help establish whether combining mechanisms provides benefits beyond those of the individual features.

These requirements can be expressed as constraints on the target force–displacement response and admissible geometry. Maurizi et al. developed GraphMetaMat to generate truss architectures for prescribed nonlinear responses while considering geometric constraints and defect tolerance [57]. Applying this approach to bioinspired coupling would require explicit representations of biological features, interfaces, and material contrast. The following section examines how process integration and structural fidelity assessment can preserve the resulting architecture and its intended deformation sequence.

## 3. Cross-Scale Fabrication Strategies for Bioinspired Mechanical Structures

The mechanical performance of a bioinspired design depends on how accurately its defining features are reproduced. Here, structural fidelity denotes the degree of agreement between the as-built structure and the intended design across relevant length scales. It encompasses geometric dimensions, topological connectivity, constituent distribution, and interfacial organization. Different fabrication methods operate through distinct structure-forming mechanisms. Biomimetic mineralization and self-assembly organize material constituents and interfaces from the nanoscale to the microscale. Freeze casting produces aligned or graded porous networks through controlled solidification. Additive manufacturing directly creates arrays, layered chambers, lattices, and TPMS architectures at larger scales. Each method offers different levels of material compatibility, structural resolution, scalability, and geometric freedom. It may also introduce defects that alter the intended load-bearing or energy-absorbing response. This section reviews these fabrication strategies according to their formation mechanisms and effective length scales, with particular attention to structural fidelity and the integration of material organization with complex geometry.

### 3.1. Bottom-Up Fabrication of Internal Material Architectures

At the smallest fabrication scales, bioinspired mechanical performance is established before the macroscopic component takes shape. Bottom-up fabrication controls the nucleation, distribution, orientation, and interfacial coupling of material constituents. Biomimetic mineralization and self-assembly are two representative routes. The former regulates the formation and deposition of an inorganic phase within an organic matrix. The latter organizes preformed building blocks through intermolecular interactions or spatial confinement. Both approaches can create internal architectures that are difficult to obtain by conventional mixing, coating, or machining.

Biomimetic mineralization reproduces the regulated formation of inorganic phases found in bone, enamel, and shells. Organic templates provide nucleation sites and control the spatial distribution of mineral growth. This process differs from simple particle filling because the inorganic reinforcement is generated in situ and coupled to the surrounding organic network [58]. Current strategies include surface mineralization, bulk mineralization, gradient mineralization, and localized directional mineralization. One route uses organic templates to guide ordered nucleation and deposition. Another route combines mineral growth with continuous polymer networks, fibrous scaffolds, or compliant matrices. Both routes can improve phase continuity, interfacial bonding, and load transfer [59].

**Figure 6:**
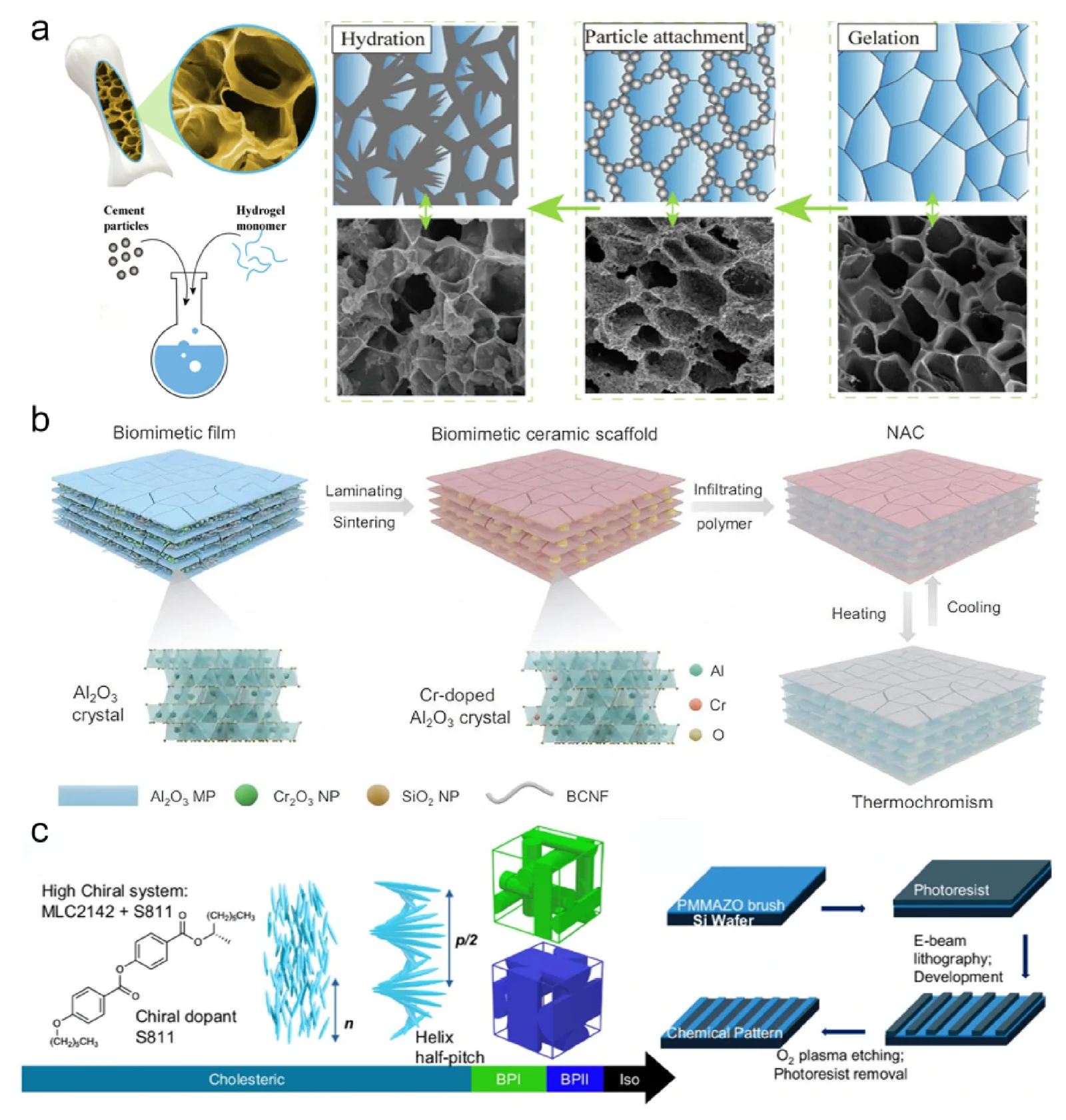
Representative bottom-up fabrication routes for bioinspired internal material architectures. (**a**) Biomineralization-inspired formation of a hydrogel-cement composite. A continuous hydrogel network is constructed first, followed by cement-particle attachment and the deposition of hydration products along the network. Adapted from [60] under CC BY. (**b**) Evaporation-induced self-assembly, lamination, sintering, and polymer infiltration used to fabricate a nacre-mimetic alumina-cyanate ester resin composite with thermochromic properties. Adapted from [64] under CC BY. (**c**) Directed self-assembly guided by chemically patterned surfaces of chiral liquid crystals into a hierarchical helical architecture analogous to the Bouligand structure. Reproduced with permission from [65], copyright 2025 Springer Nature.

Wang et al. developed a biomineralization-inspired hydrogel-cement composite with an interpenetrating organic–inorganic architecture [60]. A continuous three-dimensional hydrogel network was first formed through rapid polymerization. Cement particles then attached to the polymer network. The subsequent release of water from the hydrogel promoted cement hydration and the deposition of hydration products along the network, as shown in Figure 6a. This sequence preserved the continuous polymer framework and produced a honeycomb-like structure containing closed pores. At hydrogel contents of 2.4–13.7 wt%, the composites showed more than a 30-fold increase in flexural toughness and a 60% increase in compressive strength compared with normal cement paste. Their thermal conductivity was also reduced by 90%. These improvements were attributed to the continuous organic network, strong organic–inorganic interactions, and the ability of the porous architecture to suppress crack growth. This example shows that biomimetic mineralization can simultaneously regulate the interface, reinforcement distribution, and pore morphology.

Self-assembly organizes preformed platelets, nanofibers, polymer chains, or liquid-crystal units into ordered architectures. The process is driven by electrostatic interactions, hydrogen bonding, van der Waals forces, coordination, or capillary forces. It can therefore establish structural order from the nanoscale to larger dimensions under relatively mild initial conditions [61,62]. Nacre-inspired brick-and-mortar architectures are commonly produced through evaporation-induced self-assembly, vacuum filtration, or interfacial confinement. Jin et al. used interactions between hyperbranched poly(amido amine)s and montmorillonite platelets to fabricate nacre-inspired nanocomposite films [63]. The optimized film reached a tensile strength of 106 MPa and also exhibited gas-barrier and flame-retardant properties.

A combination of self-assembly and subsequent consolidation can extend these architectures from thin films to bulk composites. Pang et al. first assembled alumina microplatelets, silica nanoparticles, chromium oxide nanoparticles, and bacterial cellulose nanofibers into nacre-inspired films [64]. The films were laminated and sintered to form a layered ceramic scaffold. Cyanate ester resin was then infiltrated into the scaffold, as shown in Figure 6b. Silica mineral bridges connected adjacent alumina microplatelets, while chromium doping produced a solid-solution strengthening effect and reversible thermochromism. The resulting nacre-mimetic composite reached a flexural strength of approximately 290.1 MPa and a maximum fracture toughness of approximately 11.1 MPa·m^0.5^. It also provided rapid fire warning and flame-retardant behavior. This fabrication route demonstrates how the organization of building blocks can be combined with interfacial reinforcement and functional integration.

Directional self-assembly provides another route for constructing helicoidal internal architectures. Pawale et al. used chemically patterned surfaces to direct the assembly of highly chiral cholesteric liquid crystals [65]. Electron-beam lithography and plasma etching produced alternating surface-anchoring regions. The liquid crystal was then confined between the patterned substrate and a polymer-brush-coated surface. Nanoscale anchoring patterns directed the formation of microscale hierarchical helices with alternating left- and right-handed twists, as shown in Figure 6c. The resulting architecture was analogous to the rotational organization of the Bouligand structure. It exhibited anisotropic and reversible deformation together with a tunable optical response. Li et al. also reconstructed the chiral nematic organization of cellulose nanocrystals through water-assisted nanofiber sliding and hydrogen-bond reformation [66]. The resulting cellulose photonic hydrogel achieved a stretchability of more than 950% and a toughness of up to 155.5 MJ/m^3^. It also showed reversible mechanochromic behavior.

Bottom-up fabrication provides high precision in controlling phases, interfaces, and constituent orientation. However, this precision is accompanied by strong process sensitivity. Mineralization depends on reaction kinetics, ion transport, and diffusion distance. Self-assembly depends on dispersion stability, solvent removal, surface anchoring, and confinement. Small disturbances can produce incomplete mineralization, platelet misalignment, weak interfaces, or discontinuous helical domains. Most existing products therefore remain limited to films, fibers, or relatively small bulk specimens [67]. The central challenge is to preserve constituent orientation and interface quality throughout thick and geometrically complex components. Solidification-directed processing provides a practical route toward larger dimensions. A moving phase boundary can organize suspended building blocks over longer distances and convert local interactions into continuous aligned porous architectures.

### 3.2. Freeze Casting of Directional and Graded Architectures

Freeze casting, also known as ice templating, extends constituent organization from local interfaces to continuous macroscopic domains. A well-dispersed suspension or solution is exposed to a controlled thermal gradient. As the solvent crystals advance, suspended particles, platelets, fibers, or polymer chains are rejected and concentrated between neighboring crystals. Sublimation removes the frozen solvent and leaves a negative replica of the crystal morphology. The porous green body is subsequently crosslinked, sintered, or infiltrated to obtain the final material. This moving solidification front can produce aligned pores and layered walls over much larger distances than spontaneous self-assembly [68,69,70].

The resulting architecture is governed by the coupled processes of crystal nucleation, crystal growth, and constituent redistribution. The thermal gradient controls the growth direction, while the freezing-front velocity strongly affects pore size and lamellar spacing. Solids loading determines the overall porosity and wall thickness. Particle size, suspension viscosity, additives, and mold geometry also affect whether suspended constituents are rejected or engulfed by the growing crystals. Unidirectional freezing usually produces parallel lamellar or tubular channels. Bidirectional freezing introduces transverse and longitudinal thermal gradients to form long-range ordered lamellae. Radial and multidirectional freezing can further generate concentric pores, graded channels, and interconnected porous networks. These capabilities have enabled the fabrication of architectures inspired by nacre, bone, wood, and other anisotropic biological materials [71]. Time-resolved X-ray tomoscopy has also made it possible to observe crystal growth and constituent redistribution in situ, which supports a more direct connection between freezing conditions and the final architecture [68].

For layered load-bearing composites, bidirectional freeze casting provides better long-range alignment than conventional unidirectional freezing. Meng et al. combined bidirectional freeze casting with pressure infiltration to fabricate nacre-inspired B_4_C/2024Al composites [72]. Freeze casting first produced an aligned ceramic scaffold. Molten aluminum was then infiltrated into the lamellar pores to form alternating hard and ductile phases. Under dynamic compression, the composite containing 40 vol% B_4_C reached a compressive strength of 1088.6 MPa at a strain rate of 3000 s^−1^. The composite containing 20 vol% B_4_C achieved a maximum dynamic strain energy density of 133.08 MJ/m^3^. Increasing the ceramic content improved the compressive strength but reduced the deformation capacity. The dynamic response was governed by crack deflection and branching within the ceramic layers, together with plastic deformation and localized shear in the aluminum matrix. These mechanisms allowed the ordered lamellar architecture to combine load transfer with impact-energy dissipation.

**Figure 7:**
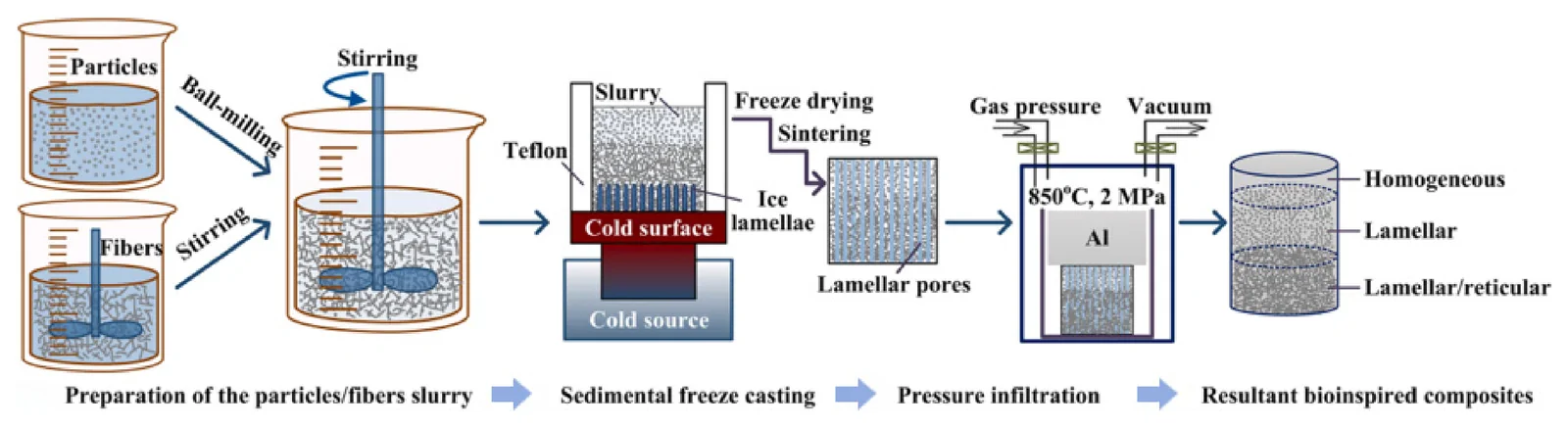
Sedimentation-assisted freeze casting and pressure infiltration for the fabrication of a bioinspired graded metal-ceramic composite. The structural gradient produces a hard bottom region, a tough intermediate region, and a softer upper region. Reproduced with permission from [73], copyright 2025 Elsevier.

Gradient formation can also be introduced by coupling sedimentation with directional solidification. Yang et al. used sedimentation-assisted freeze casting and pressure infiltration to fabricate a graded Al/(Al_2_O_3_ + mullite) composite [73]. Differences in the settling and redistribution behavior of particles and fibers generated a slurry with a spatially varying composition before complete solidification. Directional ice growth then converted this compositional variation into homogeneous, lamellar, and lamellar-reticular regions. After freeze-drying and sintering, molten aluminum was introduced through pressure infiltration, as shown in Figure 7. The resulting composite exhibited a hard bottom region, a tough intermediate region, and a softer upper region. This approach shows that freeze casting can regulate both pore orientation and material distribution. It therefore provides a practical route for transferring biological gradients into synthetic metal-ceramic composites.

Freeze casting is compatible with ceramics, metals, polymers, and hybrid material systems. Changes in the temperature field and mold configuration can generate parallel, radial, concentric, graded, or hierarchical pores. Subsequent sintering or infiltration converts these porous templates into load-bearing frameworks or multiphase composites. The aligned walls provide directional stiffness and load transfer, while the pore network and secondary phase contribute to crack control and energy dissipation. This combination makes freeze casting particularly suitable for anisotropic porous structures and graded protective materials [74].

However, freeze-cast architectures remain sensitive to nonuniform heat transfer, ice-lens formation, drying cracks, sintering shrinkage, and incomplete infiltration. For unidirectional freezing, thermally insulating mold walls and a conductive cooling base help limit lateral heat loss. Temperature monitoring and programmed cooling can regulate freezing-front velocity and reduce unintended variations in pore spacing and orientation. Slurry solids loading, dispersant dosage, and binder content should be optimized together to maintain stable particle redistribution and sufficient green strength [70]. Freeze-drying temperature and pressure should be controlled to prevent partial thawing and scaffold collapse. Controlled binder removal and sintering schedules can reduce cracking, while measured directional shrinkage should guide dimensional compensation. For infiltrated composites, pore connectivity and wetting should be considered when selecting vacuum or pressure assistance to improve filling. Post-infiltration micro-computed tomography and cross-sectional microscopy should assess unfilled regions, residual pores, and interfacial gaps. These observations can guide adjustments to infiltration temperature, pressure, and duration. Together, these measures help preserve the load paths and deformation mechanisms established by the porous template. Mold geometry and heat-flow direction still limit the fabrication of internal features with abrupt geometric variations, motivating the use of digitally controlled additive manufacturing.

### 3.3. Additive Manufacturing of Complex Bioinspired Architectures

Freeze casting creates directional order through controlled solidification, but its attainable geometry remains coupled to the growth of the solvent phase. Additive manufacturing provides a different route. It converts digital models into physical structures through sequential material deposition, selective curing, or localized melting. This approach enables complex internal topologies, spatial gradients, and integrated architectures that are difficult to produce by conventional methods [75,76,77,78]. The processes most relevant to bioinspired mechanical structures include material extrusion, vat photopolymerization, and powder bed fusion [79].

Material extrusion forms each layer by dispensing feedstock through a nozzle. In FDM, thermoplastic filaments are melted, deposited, and solidified layer by layer. Direct ink writing deposits viscoelastic inks that retain their shape through shear-thinning and yield-stress behavior [80,81]. These methods are compatible with polymers, particle-filled inks, and composite formulations. Their structural fidelity is nevertheless constrained by nozzle diameter, bead geometry, interlayer bonding, and path-dependent anisotropy.

**Figure 8:**
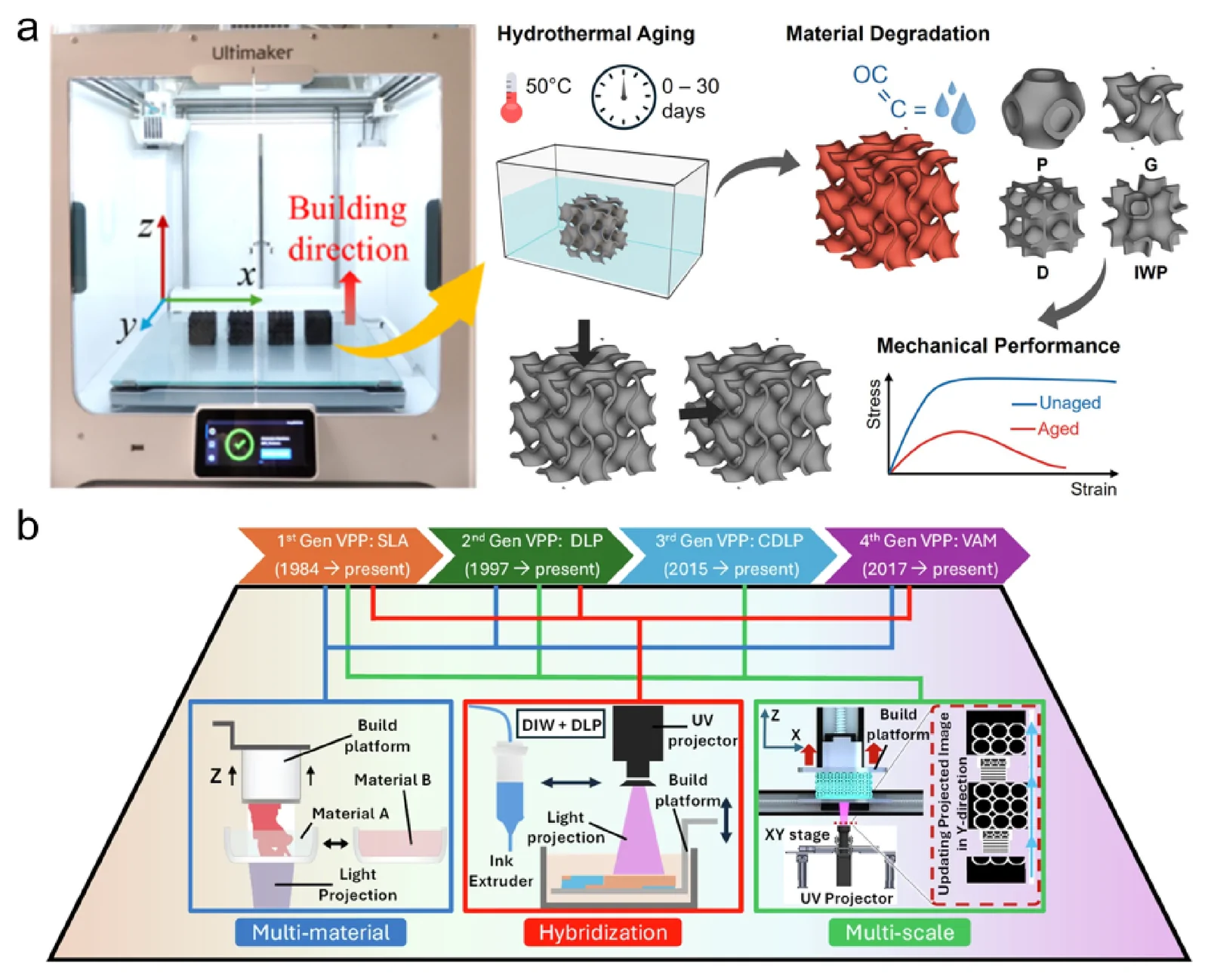
FDM and digital light processing additive manufacturing routes for complex bioinspired mechanical architectures. (**a**) FDM, hydrothermal aging, and compression testing of polylactic acid I-Wrapped Primitive, Primitive, Diamond, and Gyroid TPMS lattices. Reproduced with permission from [82], copyright 2026 Elsevier. (**b**) Development of vat photopolymerization from stereolithography and digital light processing toward continuous digital light processing and volumetric additive manufacturing. Parallel developments include multimaterial, hybrid, and multiscale fabrication. Reproduced with permission from [87], copyright 2024 Springer Nature.

Li et al. used FDM to fabricate polylactic acid TPMS lattices with Primitive, Gyroid, Diamond, and I-Wrapped Primitive topologies. The specimens were immersed in water at 50 °C and then tested under compression in different loading directions, as shown in Figure 8a [82]. After 18 days of hydrothermal aging, the Gyroid and Diamond lattices retained 96% and 94% of their initial peak stresses, respectively. All four topologies showed pronounced degradation after 30 days. These results demonstrate that topology can delay performance loss but cannot eliminate aging of the constituent polymer. Wall thickness and build orientation also affect the compressive response and energy absorption of FDM-printed Gyroid lattices [83].

Direct ink writing extends material extrusion to ceramic and metallic systems. del-Mazo-Barbara et al. printed calcium phosphate inks with a high solids loading into hydroxyapatite Gyroid, Diamond, and Schwarz architectures. The interconnected concave channels promoted SaOs-2 cell adhesion, proliferation, and mineralization [84]. Putra et al. used an iron–eggshell composite ink to produce scaffolds with approximately 70% porosity. The structures withstood three million loading cycles in simulated body fluid and exhibited a degradation rate of 0.11 mm year^−1^ [85]. These studies show that ink rheology and post-deposition consolidation are as important as the prescribed geometry.

Vat photopolymerization selectively cures a liquid photopolymer through patterned light exposure. Stereolithography and digital light processing can reproduce finer features and smoother curved surfaces than filament-based extrusion [86]. Recent developments have expanded these processes from single-resin printing toward material switching, hybrid deposition, continuous printing, volumetric additive manufacturing, and multiscale fabrication. This progression is summarized in Figure 8b [87]. These capabilities are valuable for TPMS structures because their mechanical response depends on continuous surfaces and accurate wall thickness. However, resin viscosity, light scattering, cure depth, post-curing shrinkage, and the removal of uncured resin can limit fidelity within narrow internal channels.

Mancini et al. fabricated DLP-printed Gyroid lattices with relative densities ranging from 0.20 to 0.65. Their elastic modulus, compressive strength, and energy absorption increased with solid volume fraction. Cell size also affected the deformation response [88]. Vat photopolymerization has also been combined with inverse design to create TPMS scaffolds whose effective mechanical properties approach those of cortical bone [89]. These results demonstrate the resolution advantage of light-based processes. They also show that the properties of cured polymers and ceramics must be considered together with geometric design.

Alongside material extrusion and vat photopolymerization, powder bed fusion provides a complementary additive manufacturing route for producing bioinspired structures with complex internal geometries. This process selectively consolidates successive powder layers using a laser, an electron beam, or thermal energy. Laser powder bed fusion and electron beam powder bed fusion are particularly suitable for high-strength metallic lattices. The surrounding powder supports many overhanging features during fabrication. However, it can become trapped within small or tortuous channels. Surface roughness, lack-of-fusion porosity, residual stress, and distortion of thin walls remain important limitations. Waqar et al. fabricated Ti_6_Al_4_V octet lattices with uniform and graded volume fractions by laser powder bed fusion. The most pronounced uni-graded and bi-graded vertical configurations increased compressive strength from approximately 160 to 200 MPa. They also increased volumetric energy absorption by 91% and 101%, respectively [90].

**Figure 9:**
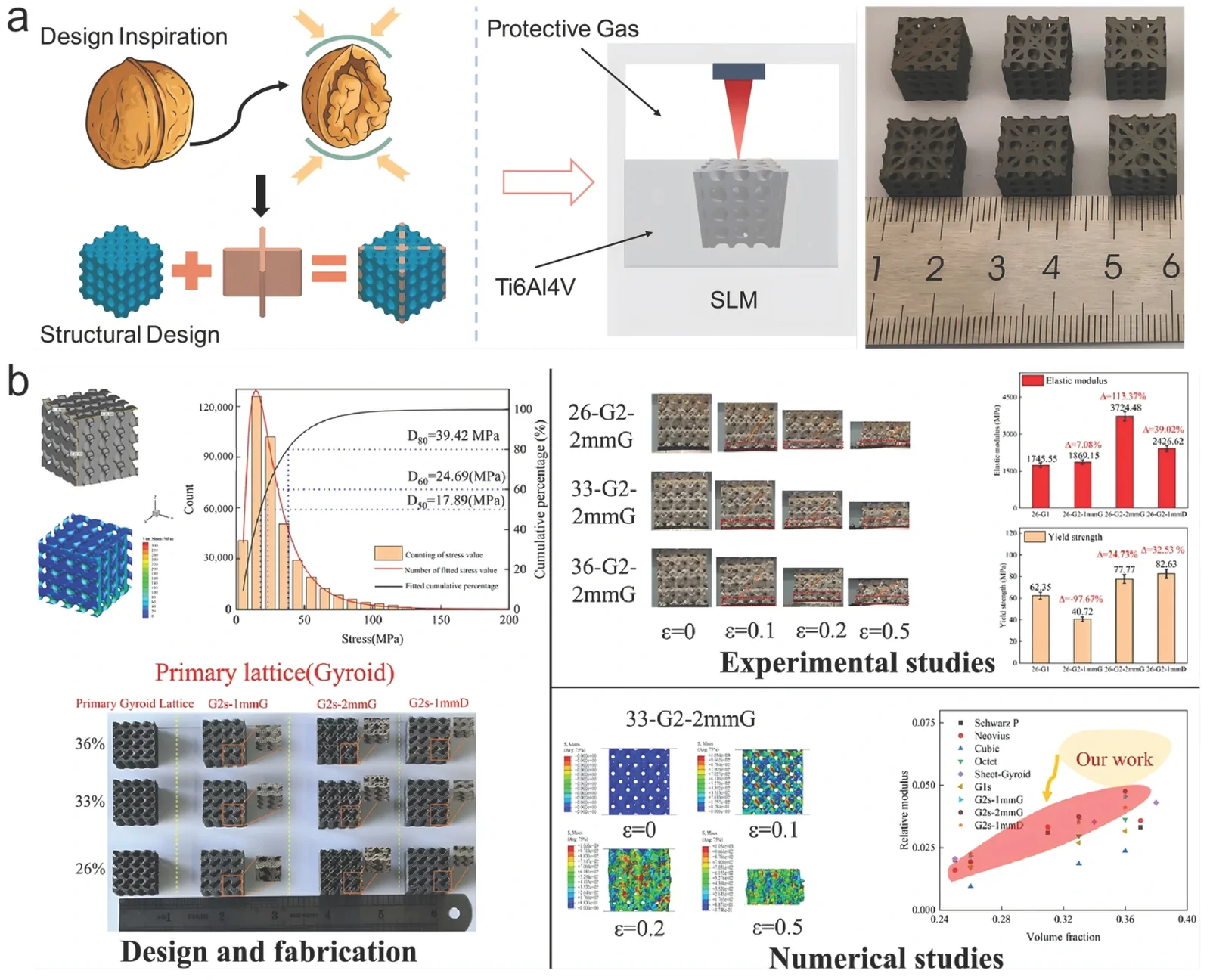
Representative powder bed fusion additive manufacturing routes for complex bioinspired mechanical architectures. (**a**) Walnut-shell-inspired functionally graded Diamond TPMS lattices fabricated from Ti_6_Al_4_V by selective laser melting. The designs contain linear and symmetric gradients in plate thickness and relative density. Reproduced with permission from [91], copyright 2025 Elsevier. (**b**) Stress-field-driven design, fabrication, compression testing, and numerical analysis of multilevel TPMS lattices. Secondary Gyroid or Diamond features are embedded within a primary Gyroid architecture. Reproduced with permission from [92], copyright 2026 Elsevier.

Bioinspired gradients can further coordinate local stiffness and progressive collapse. Xiao et al. translated the layered resistance of a walnut shell into functionally graded Diamond TPMS lattices. The Ti_6_Al_4_V specimens were produced by selective laser melting, which is a laser powder bed fusion process, as shown in Figure 9a [91]. A symmetric reverse gradient in relative density increased energy absorption by 161.60% compared with the uniform design. This improvement was associated with progressive collapse and reduced localization of deformation. Wang et al. introduced a stress-field-driven strategy for multilevel TPMS lattices. Secondary Gyroid or Diamond features were embedded in selected high-stress regions of a primary Gyroid architecture. The workflow included stress-field analysis, geometric construction, laser powder bed fusion, compression testing, and numerical simulation, as illustrated in Figure 9b [92]. The multilevel design increased energy absorption by up to 42.5% and promoted a more stable layer-by-layer collapse. Multi-topology hybrid lattices provide another route to broaden the attainable ranges of load-bearing capacity and energy absorption at relative densities between 20% and 40% [93].

Each additive manufacturing route introduces characteristic defects that can alter load transfer and collapse behavior. In thermoplastic material extrusion, incomplete contact and limited polymer interdiffusion between deposited beads produce voids and weak interfaces. These defects reduce strength across deposited layers and promote interlayer separation, which can interrupt stable progressive crushing. In direct ink writing, filament distortion and cracks formed during drying or consolidation create local weak regions that can trigger premature collapse. In vat photopolymerization, excessive cure depth and light scattering can thicken walls or close small pores, changing actual relative density, stiffness, peak force, and densification behavior. Retained resin further alters the intended cavity geometry and mechanical response [94], while uneven curing and post-curing shrinkage can introduce local stiffness variations and distortion. In metal powder bed fusion, incomplete melting produces lack-of-fusion pores, partially fused particles increase surface roughness, and thermal gradients generate residual stresses and wall distortion. Pores and surface irregularities promote stress concentration and crack initiation, while distorted walls can buckle prematurely and disrupt the intended collapse sequence [95]. Excess material can also increase mass without a proportional stiffness gain, reducing specific stiffness [96]. Process selection should therefore consider how these defects affect load-path continuity, crushing stability, and resistance to damage. Their assessment requires characterization of both as-built geometry and material integrity, as discussed in the following section.

### 3.4. Cross-Scale Manufacturing Integration and Structural Fidelity

The coupling requirements discussed in Section 2.5 guide the integration of fabrication methods considered here. Process selection and structural fidelity assessment should therefore address load-path continuity, interfacial integrity, and the intended deformation sequence. Table 2 compares the fabrication routes reviewed in terms of structural scale, material compatibility, scalability, manufacturing defects, and mechanical function. Integration should be planned around critical load paths and interfaces, checking whether each operation preserves features formed earlier. Infiltration and feedstock removal require accessible pathways, while curing and thermal treatment must remain compatible with the phases and interfaces responsible for the intended mechanical response.

Process development can change these integration constraints. Sun et al.’s thin-film DLP process demonstrates how controlling resin delivery and removal can enable enclosed cavities and material transitions [94]. At smaller scales, a different limitation emerges when material and structural dimensions become comparable [97]. In nickel nanoarchitectures, concentrated nanoporosity near junctions governed deformation initiation and size-dependent strength [98]. These examples indicate that manufacturing feasibility depends on both access to internal features and the material heterogeneity generated during their formation.

Structural fidelity tolerances should consequently reflect the mechanical sensitivity of different regions. Deviations in primary load paths should be assessed against stiffness and stability requirements, while deviations in sacrificial regions should be assessed against the intended collapse sequence. As-built reconstruction and local material characterization can be incorporated into mechanical models to identify sensitive regions and guide tolerance allocation [99]. Model predictions and subsequent geometric compensation or process adjustments should be verified experimentally. This assessment connects manufacturing accuracy to the preservation of specific mechanical mechanisms across structural scales.

## 4. Conclusions and Outlook

This review has connected four design routes for bioinspired mechanical structures, including internal material architectures, macroscopic load-bearing configurations, multilayer energy absorbers, and internal lattice architectures. Their mechanical performance depends on the coordination of material distribution, load paths, and deformation sequences. The reviewed studies indicate that effective bioinspiration requires geometric and topological features to be connected to their governing mechanical functions [100,101].

For load-bearing structures, continuous stress transfer and resistance to instability are essential, with spatial heterogeneity providing additional control over stress distributions [102]. For energy absorbers, architectural organization regulates the progression of deformation, while material-level toughening can improve resistance to impact damage [103]. Achieving both functions requires compatibility between constituent behavior and structural response. Complementary fabrication processes can connect fine material organization with complex component geometries [100], but their effectiveness must be assessed through the mechanical behavior of the as-built structure. Fabrication quality therefore forms part of the relationship between bioinspired design and engineering performance.

Future research should establish quantitative relationships between biological features and engineering functions. Phase orientation, interface waviness, characteristic length ratios, gradient distributions, connectivity, and load-path redundancy provide useful descriptors for systematic comparison and data-driven design [104]. Building on the inverse-design approaches discussed in Section 2.5, these descriptors should be translated into explicit design constraints, and the sensitivity of the resulting mechanical response to material properties and manufacturing deviations should be evaluated. Optimization should jointly consider service-load requirements, initial peak force, specific energy absorption, deformation stability, and performance variability under realistic loading conditions.

Design and fabrication should also be integrated through measurable feedback. Minimum feature size, build orientation, support requirements, and dimensional deviations should be incorporated into structural optimization before fabrication. Advances in electron microscopy, X-ray imaging, and opto-acoustic characterization provide opportunities to observe deformation and failure across scales and assess the mechanisms represented in numerical models [105]. Simulation-in-the-loop additive manufacturing has demonstrated that image-based defect detection can update structural models during fabrication and support real-time quality assessment [106]. Further development should establish when measured deviations require process correction and whether the resulting adjustments improve mechanical performance and reproducibility across successive builds. The accuracy of these systems should be evaluated together with their computational and manufacturing costs.

For multimaterial and hybrid structures, interface durability, processing compatibility, and scalability remain major challenges. Functional gradients encoded within extrusion feedstocks [107] and synchronized control of multifunctional printheads [108] provide routes to more complex material distributions. Their engineering assessment should include multiaxial loading, cyclic deformation, repeated impact, and environmental exposure. Shape recovery should be distinguished from recovery of stiffness, strength, and energy absorption capacity, particularly when damage accumulates during repeated use. Consistent benchmarking across structural sizes, fabrication processes, and defect distributions is needed to establish reliable operating conditions and support the transition from laboratory demonstrations to engineering applications.

## Figures and Tables

**Table 1:** Comparison of representative design strategies, mechanical mechanisms, and performance for bioinspired load-bearing and energy-absorbing structures.

Design Route	Biological Model	Material System	Dominant Mechanical Mechanism	Key Mechanical Performance	Limitations and Comparison Boundaries	Ref.
Material microstructural organization	Nacre and spongy bone	High-Entropy Carbides	Stress redistribution through a continuous network, plastic deformation, crack bridging, and interfacial crack deflection.	Fracture initiation toughness of 12.5 ± 1.5 MPa·m^0.5^ and flexural strength of 613 ± 52 MPa at room temperature. Approximately 97% strength retention at 1300 °C.	Excessive soft-phase content can create preferential crack paths.	[29]
	Root–soil interlocked interface	Carbon-fiber-reinforced polymer-bonded composite	Mechanical anchoring and improved interfacial bonding enhance stress transfer and suppress debonding.	Tensile strength of 7.64 MPa and fracture strain of 0.2%.	Chemical treatment may damage fiber strength.	[30]
Macroscopic array architectures	Bird-of-paradise stem	Chopped carbon-fiber-reinforced polyamide	Diaphragms constrain wall displacement, promote distributed plastic hinges, and separate local force peaks.	Specific energy absorption reached 12.838 ± 0.178 J/g.	Performance is sensitive to dimensional accuracy and diaphragm placement.	[35]
Layered and graded architectures	Cork cell walls	Multilayer cellular composites containing alternating hard and soft phases	Phase contrast redistributes strain and promotes progressive damage. The soft phase stores and releases elastic energy.	Shape recovery demonstrated after compressive deformation up to 40%.	Difficulties in coordinating strength and energy absorption.	[39]
	Cuttlebone	Photopolymer resin	Spatial variation in wall geometry regulates local buckling and the progression of collapse.	Specific energy absorption reached 8.859 J/g.	High requirements for structural dimensions.	[41]
Internal lattice and TPMS architectures	Sea glass sponge skeleton	Thermoplastic polymers	Diagonal reinforcement shortens unsupported strut lengths and redistributes stress, delaying localized buckling.	Modulus of resilience reached 6.8 × 10^6^ J/m^3^.	High requirements for structural dimensions, and thin regions are sensitive to printing defects.	[48]

**Table 2:** Comparison of fabrication strategies for bioinspired mechanical structures.

Fabrication Strategy	Characteristic Scale	Compatible Materials	Scalability Constraints	Main Manufacturing Defects	Structural Fidelity Indicators	Suitable Mechanical Functions
Biomimetic mineralization	Nano- to microscale	Mineral-polymer and mineral-hydrogel composites	Reaction uniformity and ion transport in thick sections	Uneven mineralization and discontinuous interfaces	Mineral distribution and interface continuity	Load transfer and damage resistance
Self-assembly	Nano- to microscale	Platelet composites, cellulose, polymers, and liquid crystals	Maintaining structural order in thick or large-area specimens	Misalignment, aggregation and domain discontinuities	Constituent alignment and domain continuity	Directional reinforcement and interfacial energy dissipation
Freeze casting	Microscale pores and lamellae	Ceramics, metals, polymers, and hybrids	Thermal-gradient control and freeze-drying throughput	Drying cracks and nonuniform shrinkage	Pore-size distribution and lamellar alignment	Directional load bearing and crack deflection
FDM	Submillimeter to millimeter	Thermoplastics and fiber-filled composites	Deposition time and unsupported-feature stability	Interlayer voids, weak bonding and warping	Wall-thickness and relative-density deviations	Lightweight load bearing and crushing energy absorption
Direct ink writing	Submillimeter to millimeter	Polymer, ceramic, metallic, and composite inks	Ink stability, drying, and consolidation	Filament distortion, drying cracks and residual pores	Filament dimensions and consolidation shrinkage	Porous structural support and tailored compliance
Photopolymerization routes	Microscale to millimeter	Photopolymers and photocurable composites	Build area, resin handling, and post-processing	Overcuring, trapped resin; shrinkage	Geometric accuracy	Stiffness tuning and controlled cellular collapse
Metal powder bed fusion	Submillimeter to millimeter	Printable alloys	Build volume, thermal control, and powder removal	Fusion defects; rough surfaces and residual-stress distortion	Geometric accuracy	High-strength load bearing and plastic energy absorption

## Data Availability

The original contributions presented in the study are included in the article; further inquiries can be directed to the corresponding author.
